# Supplementary material for: Spatial Distribution of Cortical Output Zones Affecting Combinations of Forelimb Muscles in the Monkey
Source: bioRxiv. 2026 Jun 30:2026.06.24.731406. Preprint. [Version 1] doi: 10.64898/2026.06.24.731406 (PMC13345131; doi:10.64898/2026.06.24.731406)
Supplement: Supplement 1 [file NIHPP2026.06.24.731406v1-supplement-1.pdf]

bioRxiv preprint doi: https://doi.org/10.64898/2026.06.24.731406; this version posted June 30, 2026. The copyright holder for this preprint (which was not certified by peer review) is the author/funder, who has granted bioRxiv a license to display the preprint in perpetuity. It is made available under a CC-BY 4.0 International license.

Supplementary Information

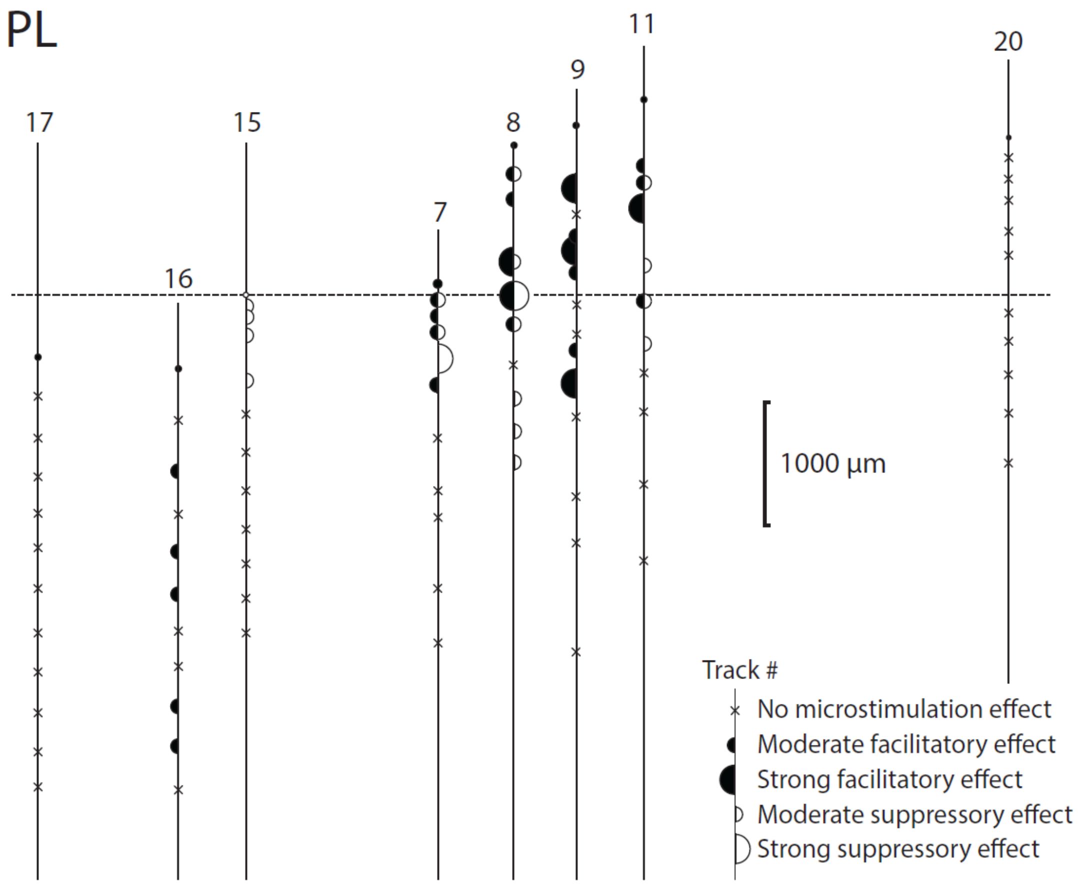

FIG. S1. Flattened map of poststimulus effects in muscle PL.

bioRxiv preprint doi: https://doi.org/10.64898/2026.06.24.731406; this version posted June 30, 2026. The copyright holder for this preprint (which was not certified by peer review) is the author/funder, who has granted bioRxiv a license to display the preprint in perpetuity. It is made available under a CC-BY 4.0 International license.

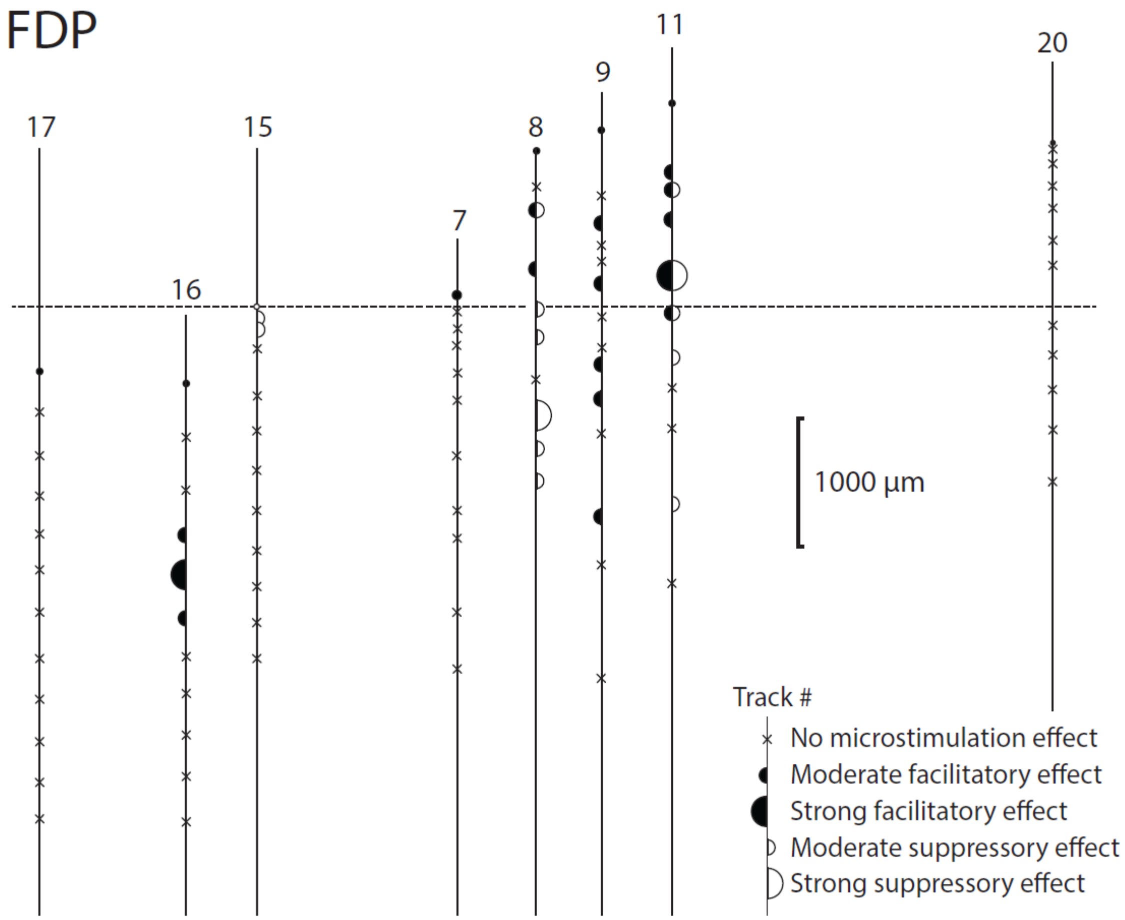

FIG. S2. Flattened map of poststimulus effects in muscle FDP.
